# Practice of district health information data for decision making and associated factors among performance monitoring team at Hadiya Zone Public Health Facilities SNNPR, Ethiopia

**DOI:** 10.1371/journal.pdig.0000552

**Published:** 2024-08-05

**Authors:** Merkineh Mekebo Madebo, Yoseph Olonjo Elitro, Bereket Birege Sundako, Adimasu Jemal Anore, Muluken Ashegire, Mengistu Lodebo Funga, Marta Tesema Lalore, Abriham Samuel

**Affiliations:** 1 Clinical Department, Hosanna College of Health Sciences, Hosanna, Ethiopia; 2 Public Department, Hosanna College of Health Sciences, Hosanna, Ethiopia; 3 Health Informatics Technician Department, Hosanna College of Health Sciences, Hosanna, Ethiopia; 4 Midwifery Department, Hosanna College of Health Sciences, Hosanna, Ethiopia; 5 Emergency Medical Technician Department, Hosanna College of Health Sciences, Hosanna, Ethiopia; Harvard University, UNITED STATES OF AMERICA

## Abstract

**Background:**

Evidence based practice is a key tool to increase effectiveness and efficiency of healthcare providers worldwide and using health facility data at all levels is vital. But, it is poorly practiced in developing countries including Ethiopia. As a result, the purpose of this study was to evaluate the level of practice of District Health Information for decision making and associated factors among performance monitoring teams in Hadiya Zone public health facilities, South Nation Nationality People Republic, Ethiopia, in 2022

**Methods:**

A facility based-cross sectional study was employed from May 3 to June 3, 2022. To obtain data, a pre-tested structured questionnaire with qualitative was employed. A multistage random sampling technique was employed to select performance monitoring team from public health facilities. Data was entered into a computer using Epi data version 4.6, and analyzed using SPSS version 25. Bivariable and multivariable analyses were used to identify determinants related to practice of district health information. For the qualitative section, thematic analysis was used.

**Results:**

The practice of district health information for decision making among performance monitoring team in this study was 48% (95% CI: [42.3, 54.1]). having standard sets of indicators [AOR = 4.055; 95% CI: (1.67, 9.86)], Being trained [AOR = 3.12; 95%CI: (1.385, 7.023)], having internet access [AOR = 3.23; 95% CI: (1.52, 6.9)], having positive attitudes [AOR = 2.667; 95% CI: (1.28, 5.56)], having low motivation [AOR = 0.202; 95% CI: (0.081, 0.504)], Sufficient skill [AOR = 3.239: 95%CI; (1.328, 8.164)] and having knowledge [AOR = 6.227; 95% CI: (2.12, 12.8)] were significantly associated with practice of District health information for decision making.

**Conclusion:**

In general, this study found that the performance monitoring team at health facilities poorly practiced district health information. It requires major improvement to provide a consistent set of indicators, training, internet access, user attitudes, motivation, and necessary skills and knowledge, as well as to raise users’ confidence in DHIS2.

## Author summary

Policy formulation, proper health management, wise resource use, monitoring and assessment of the public health situation, health care delivery, and outcomes all require accurate and timely health information. Effective use of DHIS2 information among PMT members plays a critical role in supporting evidence-based decision-making at the lower facility level. Due to the possibility provided by the ICT sector, the digitalization and expansion of HIS can support this. One of the most important tools to improve data usage in this regard is the adoption of the District Health Information System (DHIS2). The district health information system should make it simple for the managers of health facilities and the medical staff to acquire health data and make decisions while organizing and coordinating services to give high-quality medical treatment. As a result our study addressed data usage or practice at performance monitoring team level for decision making. Our finding revealed most of performance monitoring team did not used DHIS data for decision making purpose due behavioral, infrastructural and commitment problems.

## Introduction

The District Health Information Software (DHIS2) is an open-source software platform for gathering, validating, processing, reporting, and disseminating data for all health programs [1,2]. The District Health Information System generates customized reports for a variety of health indicators for local, provincial, and national health departments. It features facility-level data entry capabilities, user-defined dashboards, and a GIS interface for offline and online environments [2].

More than 67 nations in Africa, Asia, and Latin America already utilize the district health information system software known as DHIS2 to collect routine health data A country’s ability to make well-informed decisions about its health is based on its strong health information systems. For planning and tracking the progress of the SDGs, including universal health care, many nations need accurate data [2].

Study conducted in Iran showed that 10% used DHIS2 data for decision-making purposes [3]. In sub-Saharan African countries, most health data is generated at a lower health system level. However, several technical, behavioral, and organizational factors affected the practice of data that were inadequately used to inform decisions making [4,5]. In Kenya, studies show that 69.7% of data was used from DHIS2 for decision-making through case team leaders [6].

The FMOH describes the Performance Monitoring Team (PMT) as a multidisciplinary health workforce that is primarily in charge of enhancing data quality, regularly using information for decision-making, tracking progress, and enhancing the performance of health care delivery at all levels of the health system [7].

The Ethiopian FMOH adopted DHIS2 as a national electronic health management information system in January 2018 and has since used it to run programs, formulate policies, and extract and use data. The DHIS2 data consists of strategies for better decision making based on facility-based data as well as worldwide initiatives utilized to foster the culture of information utilization to accomplish sustainable development goals [8].

Specifically at the moment of data collection, FMOH had made efforts to strengthen Ethiopia’s data culture of information use. As one tool to bring about an information revolution, the FMOH unveiled the health sector reform strategy. The primary goal of this HSTP plan’s information revolution is the widespread use of health information at all levels [9]. DHIS2 is a significant instrument to help achieve the intended goals in this area. Data quality knowledge and skill gaps among the performance monitoring team are a serious problem that require ongoing work to modify data culture practices [9,10].

Various studies in Ethiopia evaluate how well health professionals use health-related information. Data quality is significantly affected by the knowledge and expertise of PMT on the reformed DHIS2 through data timeliness, completeness, and accuracy at the point of service delivery. The PMT is a key component of Ethiopia’s health system for assessing each level’s utilization of information, data quality, and health performance. The use of DHIS2 data for decision-making is directly influenced by the PMT member’s duty [11,12]. Despite this knowledge gap, there is little evidence about the level of assurance PMT members have in using District health information and the dimensions of their obligations. Therefore, the purpose of this study was to assess level of practice and associated factors among performance monitoring team in Hadiya Zone, SNNPR.

## Method and materials

### Study setting, design and sampling

From May 3, 2022 to June 3, 2022, a facility-based cross-sectional study design was used in Hadiya zone public health facilities. Hadiya zone is located in Ethiopia’s SNNRP which is 232 kilometers from Addis Ababa. There is 1 teaching comprehensive specialized hospital, 3 primary hospitals, 17 Woreda health offices, 4 town administration, 61 health facilities, and 305 health posts in the Hadiya zone. There are 2716 health practitioners from various professions in the zone.

### For Objective 1

Sample size was calculated by using single population proportion formula assuming 95% confidence interval, 5% margin of error and assuming by taking, 10% non–response rate a 45.8% Magnitude of District health information utilization for decision making in Ethiopia Ilu Aba Bora Zone of Oromia [13], Applying the formula: $n=(\frac{{\left(Z\alpha /2\right)}^{2}p(1-p)}{d^{2}})$

By all the above assumptions, the sample size was (n) is 381, 10% for the non-response rate, DE, 1.5, the final sample size (n) will be 381*1.5+38(10%) = 629.

### For objective two

#### Sample size determination for associated factors

To determine the required sample size for associated factors of DHIS2 data utilization for decision making by PMT was considered which were significantly associated with the outcome variables and by using open Epi-Info menu software online, the sample size was calculated for those selected variables as follows [Table 1].

**Table 1:** Sample size for objective two, calculated with 95% confidence level and power of 80%, RR 1:1.

Variable	Confidence level	Power	Ratio (Unexposed: Exposed)	% outcome in the unexposed group	Risk ratio	Odds ratio	% outcome in the exposed group	10% NON Resp DE 1.5	Sample size	Reference
Motivation	95%	80%	**1**	32.5%	**2.4**	3.93	76.7%	**1.5**	**146**	**16**
Competency skill	95%	80%	**1**	34.7%	**2.2**	3.83	77.0%	**1.5**	**284**	**16**
standard indicators	95%	80%	**1**	28.63%	**0.3**	3.28	7.94%	**1.5**	**366**	**35**

Finite population correction for proportions if the population is small then the sample size can be reduced slightly. This is because a given sample size provides proportionately more information for a small population than for a large population [14].

The sample size correction formula for a finite population (< 10,000) was applied since the source population is 509.


$$n=\frac{n_{o}}{1+(\frac{n_{o-1}}{N})}n=\frac{629}{1+(\frac{629_{-1}}{509})}=289$$

### Finally, the sample size was calculated after applying the correction formula. So, the final sample size was 289

In the first stage, the study district health facilities were chosen using a multistage cluster sampling technique. There are 17 districts in total, with eight districts and two town administration public health institutions chosen through simple random sampling. The samples were then distributed proportionally to each health institution based on the size of the PMT in each facility. A simple random sample methodology (lottery method) was utilized in the secondary stage of sampling to pick study participants from each specified health facility. The structured questionnaire was used to collect data utilizing quantitative and qualitative data collecting approaches. An in-depth interview with ten carefully selected key informants from hospitals and health facilities was used to acquire qualitative data. The medical director, CEO, Health center head, nursing director (Matron), nurse head, and health information focal point were all key informants. The observational checklist, document evaluations, and availability of unit, infrastructure, and resources for reporting and collecting data were used to evaluate public health facilities. To obtain extensive and in-depth information, the criteria for selecting key informants were level of stake in the organization, training, and job experience.

### Data collection method

#### Quantitative method

Data were gathered using a standardized questionnaire adapted and contextualized for our study from the PRISM tool and related literature [15–17].

A pre-tested, structured interview questionnaire adapted from the PRISM framework was utilized as the data gathering strategy and instrument. Both open-ended and closed-ended questionnaires were used. The questionnaire was written and administered in English. The questionnaire had six Socio-demographic characteristics of respondents, technical factors, behavioral, organizational factors, and utilization of District health information for decision making. The statements were rated on a five-point Scales were prepared using a Likert scale of five categories from strongly disagree to strongly agree and Yes or No type questions. The scale’s internal reliability was checked by Cronbach’s Alfa value.

### For qualitative data

To supplement or triangulate the quantitative investigation, a qualitative strategy was applied. Because there is no formula for establishing sample size in qualitative design, qualitative data was gathered through an in-depth interview with ten carefully selected key informants from hospitals and health clinics. The medical director, chief executive officer, health center head, nursing director (Matron), and DHIS2 focus were key informants. The number of key informants was determined by information saturation during data collection using an interview guide and observation of health facilities using a designed observation checklist. An observational checklist was used to collect written comments from the supervisor as well as the availability of functional computers in the unit, guidelines, and stationery items.

### Data collectors

Data was obtained via questionnaires by skilled data collectors. For data collection, eight data collectors were recruited: BSc nurses, BSc Midwives, BSc health officers, and Health informatics technicians with experience in DHIS2 activities and DHIS2 training. Collectors were chosen from out-of-study public health facilities that were not included in the study, and they were on annual leave. Two master’s holders were also appointed as supervisors to oversee daily data collection tasks.

### Operational definition

#### District Health Information System software version 2

free and open-source database application used for data collection, validation, analysis, dissemination, and presentation of aggregate and patient-based statistical data, from lower tiers to higher tiers [2].

#### Practice of District health information data for decision making

It is defined as the use of data/information from DHIS2 for analytic report production, discussion, decision/actions, target setting, information dissemination, planning, and monitoring, epidemic management, logistic management, human power supply [18]. There are a total of eleven questions and all these components have a Yes or No answer each. Percent of data utilization were then calculated for each respondent. Finally, the median score cut of the point were used to classify as “good data utilization” if the score is greater than or equal to the median score and “poor data utilization” if a score was less than the median score. The total level of DHIS2 data utilization was calculated as the average of all individual DHIS2 data utilization.

#### Performance Monitoring Team (PMT)

The PMT members are those healthcare providers serving as focal persons from their respective departments. A team that includes; the head of the facility, HMIS coordinators, case team leaders, finance, and human resource representative [9].

#### Culture of information use

The intensity of the participant’s belief regarding information used to use in the facility by staff, superiors, or head of the facility will be measured through a set of five items. All the components have a Likert scales ranging from strongly disagree to strongly agree. Finally, the mean score cut of the points was used to classify the good perceived culture of information use, and the poor perceived culture of information use [16].

#### Perceive self-competence

It is how participants perceive their competence in performing tasks related to information systems. They rated their competence in accomplishing various activities on a scale from 0–5, where 0 is “no competence” and 5 is “very strong competence”. There are a total of six items. Finally, the mean score cut of the point was used to classify as “well-perceived self-competence” if the score was greater than or equal to the median and “poor perceived self-competence” if the score was less than the median score [16].

#### Attitude

Study participants were asked a series of three questions about how they felt regarding health data. All components have a Likert scales ranging from strongly disagree to strongly agree. Finally, the mean score cut-off point was used to classify as a “favorable attitude” if the score is less than or equal to the mean score and an “unfavorable attitude” if the score is greater than the mean score [18].

#### Level of motivation

Study participants were asked a series of three questions. All components have a Likert scales ranging from strongly disagree to strongly agree. Finally, the mean score cut-off point was used to classify as “good motivation” if the score is greater than or equal to the mean score and “poor motivation” if the score is less than the mean score [16].

#### Level of knowledge

the head of the case team’s perceptions, thinking towards performing a specific activity related to DHIS. There are a total of thirteen questions and all these components have a Yes or No answer each. Good level of knowledge; respondents mean score and above, poor level of knowledge; below mean score.

#### Functional computer access

if the available computer properly functioning and are used for data recording, processing, and report writing.

#### Public health facilities

Are those facilities giving services to the public and administered by the government. In this case, includes Health centers and Hospitals.

### Data quality management

The data collectors and supervisors received two days of training on data gathering techniques, instruments, ethical considerations, and the goal of the study. Throughout the data collection period, the supervisor and lead investigators provided constant follow-up and oversight. First, data collectors presented the study’s objectives to study participants, then obtained consent and continued the study. Throughout the data collecting period, supervisors evaluated the collected data for completeness, consistency, and clarity. The lead investigator was in charge of overall oversight.

### Data processing and analysis

After data collection, data will be entered into Epi-Data version 4.6 and exported to the Statistical Package for social science for data processing and analysis version 25. Descriptive data, median, frequency, and percentage, and the result was presented by narration, tables, and charts.

Binary logistic regression was used to identify factors associated with the outcome variable. Those variables in a bi-variate analysis whose p-value is less than 0.25 (p<0.25) was taken as a candidate for multivariate analysis. The bivariate and multivariate outputs was presented in Crude Odds Ratio (COR), and Adjusted Odds Ratio (AOR), with their respective 95%CI and p-value, being less than 0.05 (p-<0.05) in the final logistic regression model was considered a statistically significant association. For the qualitative data, responses from key informants were coded, categorized, and analyzed using the thematic analysis technique manually. Transcription and translation of qualitative data was done at the same time from field notes.

### Ethical consideration

An ethical issue was approved by Hosanna Health Science College of research publication directorate Institutional Review Board (IRB). Hosanna Health Science College of research publication directorate writes a letter to the Hadiya Zone health department. Then Hadiya Zone health department writes the official letter for the Woreda health office where the study takes place. Then, communication was made with official permission is sought from the Hadiya Zone health department before data collection, Before administering the questionnaires, the objectives of the study was clearly explained to the participants, and informed voluntary written and signed consent was obtained. The study’s confidentiality was maintained throughout its execution. Participants were advised that their participation was entirely voluntary and that they might opt out of the study at any moment. All information provided by respondents was used solely for research purposes.

## Results

### Socio-Demographic Characteristics of study participants

A total of 291 PMT members were sampled, and 281 of them participated in the survey, giving a 97% response rate for the study. The majority of respondents 210 (74.7%) were between the ages of 26 and 35, with 175(63%) of residences in this category being urban. Regarding gender, 198(64.1%) of participants were male, while 180(70.5%) came from health centers. In terms of education, 154(54.78%) of study participants held a bachelor’s degree or higher. One hundred and ten (39.1%) of the participants were registered nurses. The most common response 183 (65.1%) was "less than five years of experience." **[Table 2].**

**Table 2:** Socio-demographic characteristics of participants Utilization of District health information (DHIS2) for decision making among performance monitoring team in public health facilities Southern Ethiopia, 2022 (n = 281).

Variables	Category	Frequency	Percentages (%)
Age	< = 25 years old	45	16.0
	26-35years old	210	74.7
	> = 36	26	9.3
Gender	Male	198	70.5
	Female	83	29.5
Residency	Urban	175	63.3
	Rural	106	36.7
Educational status	Diploma	103	36.7
	Bachelor’s degree and above	154	54.8
	Masters and above	24	8.5
Types of health facilities	Health centers	180	64.1
	Hospitals	101	35.9
Position in health facilities	Head	253	90.0
	Expert	28	10.0
Work Experience in years	<5	183	65.1
	> = 5	98	34.9
Field of specialty	MD/Specialist	17	6.0
	Health officer	56	19.9
	Nurse	110	39.1
	Midwife	20	7.1
	Medical laboratory	22	7.9
	Pharmacist	17	6.0
	HIT	23	8.2
	Environmental health	3	1.1
	Others	13	4.6

### Organizational Characteristics of the study participants

**Fig 1:**
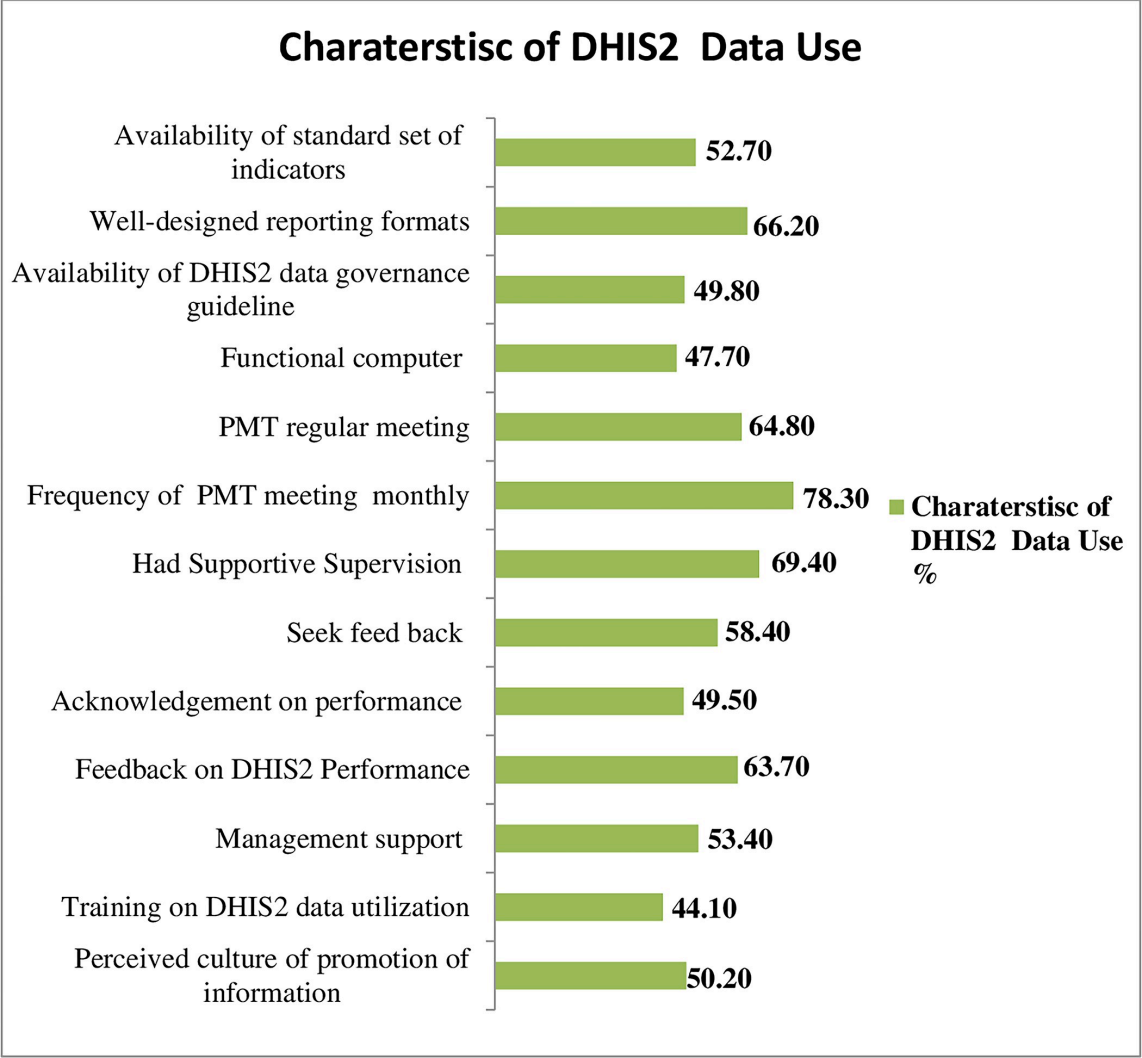
Organizational characteristics of utilization of District health information(DHIS2) for decision making among performance monitoring team in public health facilities Southern Ethiopia, 2022(n = 281).

This research revealed that around half 141(50.2%) of participants promoted data culture. Regarding DHIS2-related tasks, about 157(55.9%) of respondents said they had not received enough training. The Finding from a key informant interview supported this result. Two hundred fifty (53.4%) of participants in this study reported managerial assistance, and 139(49.5%) reported receiving praise for their strong performance. One hundred sixty four (58.4%) of responders express a desire for input. The next higher health authority regularly provides feedback to more than 179(63.7%) of responders about DHIS2 duties and encouraging supervision. One hundred ninety five (69.4%) of respondents said their boss regularly supervises them, and 182 (64.8%) of PMT had conducted meeting regularly. This is supported A key in-depth informant. The majority of research participants reported that 140(49.8%) of their teams had the DHIS2 data governance standard, and more than two thirds of them 220(76.3%) had reviewed their performance on a monthly basis and 140(49.8%) of survey participants reported having DHIS2 data governance guidelines, while 148(52.7%) of participants reported having a common set of indicators **[Fig 1]**

### Technical and Behavioral characteristics

Nearly half of participants 142(50.5%) had both online and offline DHIS2 Software for data entry and analysis, while 119(43%) of participants had no internet connectivity in their health facilities. The Finding from a key informant interview supported this result. One seventy seven (63%) of participants in the current study thought the forms used to collect and submit data were user-friendly. Less than half of 123(43.8%) of participants said the methods for gathering and reporting data were complicated. About 105(37.4%) had lack of understanding of DHIS2 related material, and 171(60.9%) of PMT Members had negative attitudes regarding DHIS2-related information. The Finding from a key informant interview supported this result.

The majority of participants 156(55.5%) had strong desire for learning about the DHI2 and 167(59.4%) had good competency skills for gathering, processing, analyzing, and interpreting data [Table **3].**

**Table 3:** Technical and Behavioral Characteristics of District health information (DHIS2) utilization for decision making among performance monitoring team in public health facilities Southern Ethiopia, 2022(n = 281).

Variables	Response	Frequency	Percentages
Availability of Friendly reporting forms	No	104	37
	Yes	177	63
Trained staff able to fill out reporting forms	No	156	55.5
	Yes	125	44.5
	Total	281	100
Availability of DHIS2 online and offline software	No	139	49.5
	Yes	142	50.5
The complexity reporting forms	Complex	123	43.8
	Not complex	158	56.2
Availability of separate office or unit for RHIS	No	86	30.6
	Yes	195	69.4
Availability of internet access	No	119	42.3
	Yes	162	57.7
Level of knowledge on DHIS2	Poor	105	37.4
	Good	176	62.6
Motivation	Poor	125	44.5
	Good	156	55.5
Attitudes	Poor	171	60.9
	Good	110	39.1
Level of competency	Poor	114	40.6
	Good	167	59.4

### DHIS2 information utilization for decision making among performance monitoring team

In this study area, the overall usage level of routine health data from DHIS2 for decision-making was good (48%)

This result showed that 218(77.6%) of PMT had used DHIS2 data for decision-making, with roughly 225(80.1%) using it to raise the competence of healthcare services. Two hundred twenty four (79.7%) of those interviewed said they use the DHIS2 data for future planning. One hundred ninety (67.6%) of PMT had been used for logistical supply. A majority of PMT 234 (84.7%) had utilized DHIS2 data to compare target with outcome.

While fewer than half of PMT 137(48.8%) indicated that they use DHIS2 data to identify and manage epidemics. More than three quarters of PMT 237(84.3%) had stated that they have the right to access DHIS2 data whenever they need it **[Table 4]**

**Table 4:** Practice of district health information (DHIS2) for decision making among performance monitoring team in public health facilities Southern Ethiopia, 2022(N = 281).

Variables	Response	Frequency	Percentages (%)
Decision making purpose	Yes	218	77.6
	No	63	22.4
Make decisions on health service quality improvement	Yes	225	80.1
	No	56	19.9
Make decisions on Medicine supply	Yes	190	67.6
	No	90	32.4
Make decisions on human resource management	Yes	190	67.6
	No	90	32.4
Have the right to Access any time when needed	Yes	237	84.3
	No	44	15.7
Day-to-day routine activity management	Yes	155	55.5
	No	126	44.5
For planning future actions	Yes	224	79.7
	No	57	20.3
For finding the root causes of the problem	Yes	179	63.7
	No	102	36.3
To compare outcome achievements with objective	Yes	238	84.7
	No	43	15.3
Community education and resource mobilization	Yes	221	78.6
	No	60	21.4
To manage epidemics	Yes	137	48.8
	No	144	51.2
For budget allocation	Yes	161	57.3
	No	120	42.7

### Factors associated with the level of RHIS for Decision making from DHIS2 among Performance monitoring team

During bivariable analysis, all variables with a p-value of less than 0.25 were entered into multivariable logistic regression analysis. In the study, variables with p-values less than 0.05 in multivariate analysis were considered statistically significant. The variables that significantly predicted practice of DHIS2 included standard set of indicators, having adequate training, having internet access, attitudes towards to DHIS2 related tasks, Motivation, skill of competency, and knowledge on DHIS2.

In multivariate logistic regression analysis, having standard sets of indicators increases DHIS2 information utilization for decision making by [AOR = 4.055; 95% CI: (1.67, 9.86)], being trained [AOR = 3.12; 95%CI: (1.385, 7.023)], having internet access [AOR = 3.23; 95% CI: (1.52, 6.9)], having positive attitudes [AOR = 2.667; 95% CI: (1.28, 5.56)], having low motivation reduces DHIS information utilization by about by 79.8% [AOR = 0.202; 95% CI: (0.081, 0.504)], sufficient skill [AOR = 3.239: 95%CI; (1.328, 8.164)] and having knowledge [AOR = 6.227; 95% CI: (2.12, 12.8)] were significantly associated with practice of DHIS2 Information for decision making **[Table 5].**

**Table 5:** Bivariable and multivariable analysis of factors associated with the level of RHIS from DHIS2 among health care providers in public Health Facilities, Hadiya Zone Southern Ethiopia 2022.

Variables	Category	RHIS utilization (n = 281)		COR (95% CI)	AOR (95% CI)
		Poor	Good	Crude	Adjusted
Residency	Urban	101	45	0.54.3, 0.881)	
	Rural	74	61	1	
Types of health facilities	Health centers	39	80	2.357(2.423,3.903)	
	Hospitals	25	100	1	
Perceived culture of information utilization	No	101	45	5.331(3.2,8.88)	
	Yes	40	95	**1**	
Management support	No	103	43	4.485(2.72,7.4)	
	Yes	47	88	**1**	
Had data governance	No	90	56	2.732(1.686,4.4)	
	Yes	50	85	**1**	
Internet access	No	104	42	**3.29(2.005,5.39)**	3.23(1.516,6.9)*
	Yes	58	77	**1**	**1**
Training on DHIS2	In adequate	100	46	**10,054(5.72,17.7)**	3.12(1.516,7.02)*
	Adequate	24	111	**1**	**1**
Had standard set of indicators	No	114	32	**10.58(6.093,18.38)**	4.055(1.668,9.856)*
	Yes	34	101	1	
Had well-designed reporting tools	No	111	31	3.344(1.986,5.630)	
	Yes	71	64	1	
Had both online and offline software computer	No	75	71	1.8(1.16,2.893)	
	Yes	50	85	1	
Separate RHIS Unit	No	122	24	4.317(2.483,7.5)	
	Yes	73	62	**1**	
Status of PMT	No	114	32	3.51(2.093,5.888)	
	Yes	68	67		
Feed back	Not regular	95	51	3.85(2.348, 6.322)	
	Regular	44	91	**1**	
Seek feedback	No	100	46	2.142(1.48,3.82)	
	Yes	64	71	1	
Acknowledge to good performance	Poor	95	51	3.852(2.348,6.322)	
	Good	44	91	1	
Trained staff able to fill out RHIS forms	No	95	51	6.52(3.84,11,07)	
	Yes	30	105	1	
Friendly formats	No	109	37	2.9(1.755,4.78)	
	Yes	68	67	1	
Complexity of reporting tools	Complex	112	34	6.373(3.777,10.58)	
	Not complex	46	89		
Level of knowledge	Poor	117	29	**5.197(3.059,8.83)**	6.23(2.122,12.9)*
	Good	59	76	**1**	**1**
Attitude	Poor	85	61	**6.13(3.556,10.6)**	2.7(1.28, 5.56)*
	Good	25	110	**1**	**1**
Motivation	Not motivated	95	51	**2.26(1.398,3.65)**	.202(0.081,0.504)*
	Motivated	61	74	**1**	**1**
Level confidence	Poor	115	31	**5.921(3.479,10.02)**	3.29(1.33, 8.16)*
	Good	52	83	**1**	**1**

Note: *Variable significant at p-value less than 0.05, 1 = reference

### Qualitative part

Interview questions were expected to be directed towards three categories of investigation: Level of utilization district health information for decision making, functional status of PMT, factors that could facilitate level of utilization and challenges to use district health information for decision making among PMT. Analysis of interview transcripts revealed key themes grouped into one of the above four categories.

One of the respondents described how the use of information in decision-making can make people feel more confident when using DHIS2 data "Information use is highly valued in our team members. This makes it possible for us to carry out our plan to use district health information effectively. We are able to calculate for this using technology that allows a person to refresh themselves with information for decision-making." Medical doctor who are 34 years old.

Another responder clarified as level of decision-making use of district health information. “Before we share information with higher levels, the performance monitoring team members in the health center review it and agree on any gaps and an action plan. Additionally, we use DHIS2 data to support the logistical needs of the service-delivery unit, which includes birth, family planning, antenatal care, and pharmacy prescription supply”. 36-year-old female key informants from a health clinic said.

Another responder provided the following description of one of the 40-year-old male Key Informants who was interviewed: "Perception of Performance motoring teams in District health information utilizations." Information utilization by PMTs was not well perceived. The ability of health workers or managers to use health information for decision-making is hampered by factors such as access to the internet, registration books, and a lack of training that is used to update knowledge.

Another person who spoke about the functional status of the PMT said "I believe that our hospital encourages PMT members to use District health information for decision-making on a daily basis until monthly performance reviews," one hospital responder said in their explanation. Additionally, we spent the majority of each month talking about the indicators with the heads of each department and comparing our catchment goals. The use of information for making decisions is now good. Although all departments had a favorable attitude about gathering regular data. Data have no meaning in nature until they are changed into action, according to our data culture motto. Male, 34 years old Chief executive officer”.

According to another reply, "Our health center performance monitoring team made evaluation on monthly basis before passing report to the next authorized district health office," the members of the group are obligated to use district health information. Female health information officer, age 26. Another person who was extensively questioned said, "In our monthly meeting, we address underperformance from the previous month. After discussing underperformance, we assess improvement and gaps. After evaluating, we make plans for closing the deficit. Since we consistently evaluate the data, we perform better! We compete in silence with the other districts in this division, and we consistently outperform them.–A 32-year-old man is the district health manager.

The majority of the participants in the in-depth interviews concurred that one of the enabling factors for their ability to use District health information was their competence and commitment of their time, resources, and efforts. "Having a consistent set of indications, training for positive attitudes, motivation, and adequate skill, as well as manager feedback and follow-up. So that I may confidently claim to be knowledgeable about using district health information for decision making.

Respondents had encountered issues like, "On behalf of our hospital, we have encountered many challenges while trying to use district health information for decision-making problems like, he connection is unstable internet access when we want data from DHIS2," which prevented them from using district health information (DHIS2) at a high level. It is difficult to enter and analyze the offline mode. It is difficult to cram in activities like data reporting and collection because of how long things take to do online. When looking for information to help us make judgments, the bad connection typically gives us problems and prohibits us from entering or seeing data because there aren’t enough computers, standardized registration log books, or human resources. a female primary health unit director, age 31.

## Discussion

The percentage of PMT members that used DHIS2 data for decision-making in this study was 48% (95% CI: [42.3, 54.1]), which is higher than the 35.75% and 45.8% and 45% found in studies done in Iran, Eastern Ethiopia’s Dire Dawa and, Illu Aba Bora Oromia region respectively [4,13,19]. This could occur as a result of the Ethiopian government’s current emphasis on the use of health information systems for decision-making and PMT’s internal positive attitude towards to information utilization [20]. The result of this study is lower than the study conducted in Ghana 77.3% [21]. The infrastructure and technological improvements may be too responsible for this. This study falls short of a Nigerian study that suggested PMTs were required to use a standard health information system was from 60–80% [4] and to the extent, very far away from WHO’s target standards to use a health information system that was targeted in 2010 which was 90% [22], and FMOH give attention to increases data utilization culture from 52%-85% [23]. This could be the number of research participants, the extent of their involvement, the accessibility of infrastructure, and the availability of resources like internet connections and other relevant electronic equipment are all potential explanations for this difference. *This result is supported by the findings from one of “A 31-year-old medical director as stated below; “On the behalf of our hospital*, *we have encountered many challenges while trying to use district health information for decision making problems like*, *he connection is unstable internet access when we want data from DHIS2*. *The offline mode is difficult to entry and analysis*. *Due to the time it takes to do tasks online*, *it is challenging to crowd in activities like collecting*, *data reporting*.

The likelihood that PMT would use DHIS2 information for decision-making was approximately four times higher among those who had a standard set of health indicators in their offices than did not have [AOR = 4.055; 95% CI: (1.67 to 9.86)]. This might be due to the presence of data sources which offer utilization of information for evidence based decision making [15]. This finding is supported by studies conducted in north Gondar [24].and Hadiya zone [25].

The good utilization of DHIS2 information for decision making among PMT was also associated with being trained [AOR = 3.12; 95%CI: (1.385 to 7.023)]. This result is supported by the study conducted in Western Amhara which showed being trained about health information was positively correlated with utilization of DHIS2 [26]. Other studies conducted in Ethiopia and Ghana stated that being trained was positively associated with utilization of DHIS2 [27,28]. The possible reason might be because usage and interpretation of data captured from training would enhance decision making and it might create skilled human resource that are confident and motivated to perform data management tasks. Additionally, training could capacitate PMTs on how to change data to information and use it.

Odds of DHIS2 information utilization of PMT who had internet access in their office [AOR = 3.23; 95% CI: (1.52 to 6.9)].

In this study, PMT with favorable attitudes were 2.7 times more likely to utilize DHIS2 data for decision making than poor attitudes towards to DHIS2 information [AOR = 2.667; 95% CI: (1.28 to 5.56)]. This study supported by the study conducted in Eastern Ethiopia and Kenya [19,29]. This is because if people think that data is useful activities or not a waste of time, they are likely to perform RHIS tasks [15]. This result matches with the study done in Kenya and Ghana [29,30]. Besides, the result is also consistent with reports of USAID and MEASURE Evaluation [31].

This result is supported by the findings from one of 34 years old male CEO “I think in our hospital currently there is good utilization for decision making. While all departments had positive attitude towards to collected routine data. Our motto on data culture is data have no value in nature without changing into action “.

Odds of DHIS2 information utilization of PMT who had low motivation reduces DHIS2 information utilization for decision making among PMT by about by 79.8% compared with respondents who had high motivation [AOR = 0.202; 95% CI: (0.081 to 0.504)]. This result is consistent with the studies conducted in Ghana [32] and East Wollega zone, Oromia region of Ethiopia [33]. This might be due to the fact that health professionals who received rewards for their good works and who are motivated to use data had always good utilization of DHIS2 for decision making. *This low motivation was supported by the Findings from one of a 40 years old male Key informants interviewed participant as stated below*: *"The perception of performance monitoring team on information use was poor*. *Access to internet*, *registration book*, *lack of training which are used for updating knowledge are hindered the health workers or managers from using health information for decision-making*.*"*

The odds of DHIS2 information utilization of PMT who had good competency were 3.24 times more likely to have good utilization of DHIS2 information for decision making when compared with respondents who had low competence on routine health information tasks [AOR = 3.24: 95%CI; (1.328, 8.164)]. This result is consistent with the study conducted in Ethiopia, Dire Dawa and Harari regional state, Kenya, and Ghana respectively [18,20,33]. It was also supported by another study [15]. The study conducted in Ethiopia also revealed similar findings [27]. Another study conducted in the western Amhara region of Ethiopia revealed that having good skills was positively associated with the utilization of DHIS [26].

But this study was inconsistent with the study conducted in Kenya which indicated that competency in RHIS task has no associations with performance of health information systems [32].

The odds of DHIS2 information utilization of PMT who had good knowledge were six times more likely to have good utilization of DHIS2 information for decision making when compared with respondents who had poor knowledge[AOR = 6.227; 95% CI: (2.12, 12.8)]. Accordingly, without processing data into information, it is not possible to utilize routine health information [9,29]. There is a need to improve information-use practices with better understanding of the health indicators and added skills on analysis and information use, and mentorship to all involved health workers [28]. This finding is supported by that of studies conducted in Western Amhara, Northern Ethiopia [26] and North Gondar zone, Ethiopia [24].

The results of a qualitative investigation also showed that the computer and data analysis skills of performance monitoring teams had a positive effect on the usage of health information. Another finding from the qualitative analysis suggested that performance monitoring teams lack motivation to use health information for decision-making due to infrastructure, such as a lack of registration logbooks, inadequate training, internet issues, the workload of health workers, a lack of computers, and inexperienced staff.

### Strength and Limitation of the study

The use of a hybrid methodology to triangulate quantitative and qualitative research was strength of this study. Another area of strength was the research used to develop proven tools from the Performance of Regular Information System Management (PRISM).

This study may have the following limitation: only a small number of significant informants were interviewed due to time and financial constraints.

## Conclusion

Decisions made by PMT based on District health information were not well practiced in the study area. Standard sets of indicators, training, internet accessibility, positive attitudes, low motivation, adequate skill, and knowledge were the most significant drivers of DHIS2 information utilization for decision making among PMT members.

The qualitative study’s findings revealed that performance monitoring teams lacked a working computer, a registration log book, adequate staffing levels, and internet connectivity. The findings also highlighted a barrier to accessing health information for decision-making, low motivation, which was associated with poor utilization. Therefore, it is strongly advised to turn data culture into action by making standard sets of indicators available, enhancing training, making internet access available, altering attitudes, and improving the skills, knowledge, and motivation of Performance monitoring teams.
